# Post-thyroidectomy tracheocutaneous fistula; A case report with literature review

**DOI:** 10.1016/j.ijscr.2020.05.055

**Published:** 2020-05-30

**Authors:** Abdulwahid M. Salih, Fahmi H. Kakamad, Imad J. Habibullah, Karzan Mohammed, Suhaib H. Kakamad, Shvan H. Mohammed, Berwn A. Abdulla, Hiwa O. Baba, Rawezh Q. Salih, Dahat A. Hussein

**Affiliations:** a Faculty of Medical Science, College of Medicine, University of Sulaimani, François Mitterrand Street, Sulaimani, Kurdistan, Iraq; b Smart Health Tower, François Mitterrand Street, Sulaimani, Kurdistan, Iraq; c Kscien Organization for Scientific Research, Hamdi Street, Sulaimani, Kurdistan, Iraq; d Iraqi Board For Medical Specialties, Sulaimani Teaching Hospital, Sulaimani, Kurdistan, Iraq

**Keywords:** Ischemic tracheal injury, Necrosis, Thyroidectomy

## Abstract

**Introduction:**

Total thyroidectomy represents one of the commonest procedures performed for thyroid diseases. The aim of this paper is to report a rare case of tracheocutaneous fistula after total thyroidectomy.

**Case report:**

A 44-year-age female presented with left side neck swelling for 2 month duration. Neck ultrasound showed a well-defined left thyroid nodule (25 × 15 × 14 mm) with features highly suggestive of malignancy, under general anesthesia total thyroidectomy was performed. On the third postoperative day, the patient came back with neck swelling especially during speaking, there was subcutaneous emphysema, wound opened with residual air leak. Under local anesthesia, the wound opened, there was 10 × 10 mm opening in the anterior aspect of trachea, a tracheostomy was inserted, the patient was sent home after decannulation with an opening in the anterior neck. Twenty days later the tracheal opening closed spontaneously.

**Discussion:**

The possibility of a tracheal wall ischemic necrosis is plausible due to cautery use. Some autopsy studies have demonstrated that small branches of inferior thyroid artery form the main blood supply of the upper segment of trachea. These fragile branches have a lateral entry point that can be damaged readily leading to ischemia and necrosis.

**Conclusion:**

Ischemic tracheal necrosis, although very rare, is possible after total thyroidectomy, minimal use of electro-cautery is advised whenever possible.

## Highlights

• Total thyroidectomy represents one of the commonest procedures performed for thyroid diseases.
• Ischemic tracheal necrosis an extremely rare complication of thyroidectomy.
• In this paper is to report a rare case of trachea-cutaneous fistula after total thyroidectomy.

## 1 Introduction

Total thyroidectomy represents one of the commonest procedures performed for thyroid diseases, whether benign or malignant. It is relatively a safe procedure in experienced hands, with an overall complication rate of approximately 5% when proper techniques are used [1,2]. Vocal fold paresis or paralysis, hematoma, hypocalcemia, hypoparathyroidism and wound infections represent the common postoperative complications. Tracheal injury on the other hand is rare. During thyroidectomy, the trachea is susceptible to injury, however it is often immediately recognized when any perforation or laceration occurs and is repaired promptly, resulting in little morbidity. The problem arises when the injury is unrecognized or there is delayed rupture due to tracheal ischemia, in which the presentation can be delayed up to 2 weeks postoperatively [1].

Here we present a case who, 3 days after total thyroidectomy, developed tracheal necrosis and rupture and discuss the workup, treatment and preventative measures with brief literature review. The work has been reported in line with SCARE guideline [3]

### 1.1 Patient information

A 44-year-age female from urban area presented with left side neck swelling for 2 month duration which was growing slowly. Past medical and past surgical history was unremarkable. She was neither smoker nor drinker.

### 1.2 Clinical examination

There was a left side neck mass, firm, non-tender, with smooth surface and mobile with deglutition. No lymphadenopathy.

### 1.3 Investigation

Neck ultrasound showed a well defined left thyroid nodule (25 × 15 × 14 mm) with features highly suggestive of malignancy, fine needle aspiration cytology (FNAC) under ultrasound guide was done, the result showed papillary thyroid carcinoma, betheda 6, in a background of lymphocytic thyroiditis. Thyroid function tests, serum calcium, serum thyroglobulin and Thyroperoxidase (TPO) antibody titers were normal. Both vocal cords were visualized and normal in texture, color and mobility. She was prepared for total thyroidectomy.

### 1.4 Intervention

Under general anesthesia in supine position through a collar incision, total thyroidectomy was performed with preservation and exploration of both recurrent laryngeal nerves and all parathyroid glands. Left side level 6 lymph node dissection was performed as well, the surgery was uneventful and patient discharged after 24 h.

### 1.5 Follow up

**Fig. 1:**
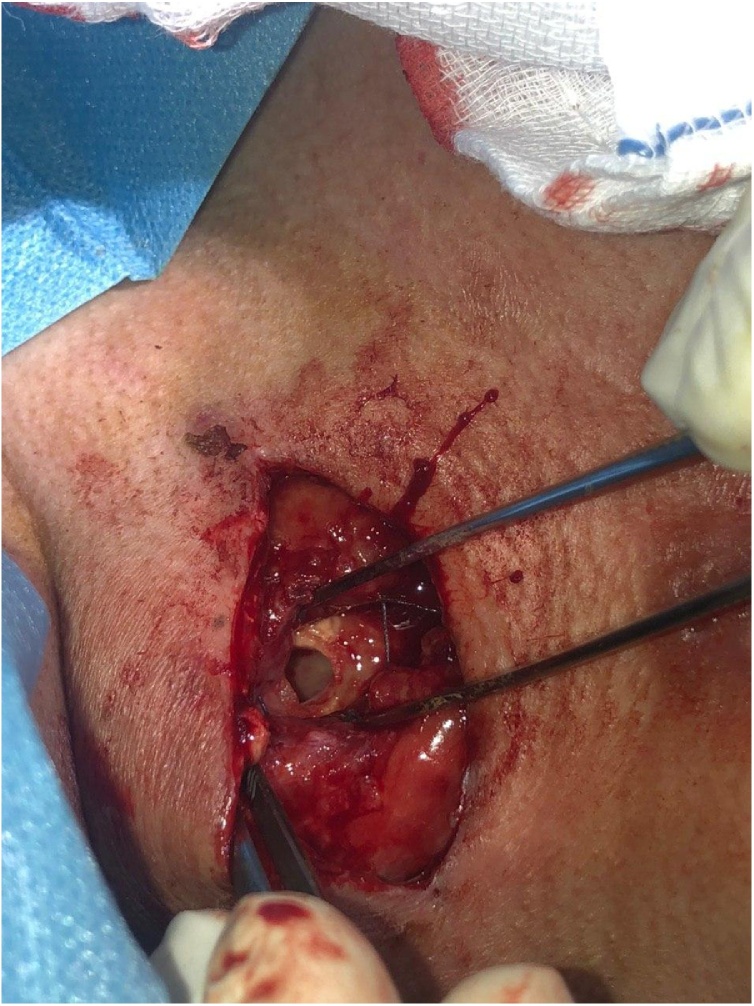
Intraoperative findings showing 10 × 10 mm hole in the anterior aspect of the trachea.

On the third postoperative day, the patient came back with neck swelling especially during speaking, there was subcutaneous emphysema, wound opened with residual air leak confirming tracheocutaneous fistula. Under local anesthesia, the wound opened, there was 10 × 10 mm opening in the anterior aspect of third tracheal ring (Fig. 1), a 7.5 French plastic tracheostomy was inserted after refreshing the age. Decannulation was done 5 days later, the patient was sent home with an opening in the anterior neck, she was advised to do daily dressing. Twenty days later the tracheal opening closed spontaneously and the skin opening was closed with a single stich.

## 2 Discussion

The disruption of the airway has been reported with chemotherapy and radiotherapy, tracheal necrosis may occur as a secondary effect with subsequent spontaneous perforation [2]. Prolonged intubation has also been reported as an iatrogenic cause for tracheal disruption, either as a result of direct traumatic injury or continued pressure leading to tissue ischemia and necrosis [4]. However, only few reports can be seen in the literature describing tracheal necrosis after thyroidectomy [1]. The authors postulate the injury to be related to excessive use of cautery on trachea or around it. Lateral dispersion of heat is a well-known phenomenon of electro-coagulation, with an inherent risk to damage surrounding tissue, trachea in this case, when used to control bleeding [5]. The injury may not be apparent and go unnoticed. As a consequent, necrotic debris and localized hematoma forms, acting as a nidus for superadded bacterial infection with necrosis as a possible outcome. To avoid injury to recurrent laryngeal nerve during thyroidectomy procedure, we tried to use electro-cautery as minimal as possible [1].

In light of the fact that surgical resection in patients with metastatic medullary thyroid cancer is still the best option providing the highest chance of survival and palliation in the long term, our patient underwent total thyroidectomy and level six neck dissection [6].

The possibility of a tracheal wall ischemic necrosis is plausible due to cautery use. Some autopsy studies have demonstrated that small branches of inferior thyroid artery form the main blood supply of the upper segment of trachea [7]. These fragile branches have a lateral entry point that can be damaged readily leading to ischemia and necrosis. Similar findings have been reported in cases of esophagectomy whom underwent dissection of cervical and upper mediastinal lymph nodes, showing tracheobronchial mucosal ulceration and necrosis [8]. As a part of chest physiotherapy, coughing was advised, which may have worsened the condition once the air leak was established.

A leak from trachea is potentially life threatening, due a rapid and expanding surgical emphysema. Fiberoptic bronchoscopy is diagnostic [9,10]. At the earliest sign, neck exploration is of paramount importance and an early tracheostomy is lifesaving. During the thyroidectomy an unintended minor injury to the trachea may have occurred in our patient that went unnoticed. The potential ischemia due to dissection as explained above, coupled with this minor injury could have led to necrosis and perforation of trachea in the post-operative period. Unfavorable outcome was averted by direct re-exploration and tracheostomy. In the current case, the patient was stable, there was already opening in the wound and fistula established, just simple enlargement of the wound make the tracheostomy tube insertion possible.

Re-exploration with primary closure of the tracheal opening is possible especially when the injury is small. Edward Damrose and John Damrose published their experience with a 20-year old male. He was a known case of Grave’s disease with exophthalmos and hyperthyroidism. They performed total thyroidectomy using both harmonic scalpel and electro-cautery. The operation was uneventful apart from hypocalcaemia which was controlled well with supplementation. Seven days after the operation, the patient presented to emergency room with hypoxia and diffuse surgical emphysema. He was intubated and re-explored, they found a 1 × 2 cm tracheal opening at the anterior surface of the trachea. They primarily closed the opening and the patient recovered well [1].

Chauhan and associates reported a 65-year-old patient underwent total thyroidectomy with bilateral neck dissection, the patient has uneventful postoperative course until the seventh postoperative day when he presented with subcutaneous emphysema involved the face and upper chest and deteriorated rapidly developing into acute respiratory distress. The patient was taken to the operating room and re-explored. There was a small opening (5 mm) in the trachea which was fashioned to adopt a 7.5 French tracheostomy tube. The patient decannulated five days later and the tracheostomy closed in 14 days [6].

In conclusion, Ischemic tracheal necrosis, although very rare, is possible after total thyroidectomy, minimal use of electro-cautery is advised whenever possible.

## Conflicts of interest

There is no conflict to be declared.

## Sources of funding

No source to be stated.

## Ethical approval

Approval is not necessary for case report in our locality.

## Consent

Consent has been taken from the patient and the family of the patient.

## Author contribution

Abdulwahid M. Salih: Surgeon performing the operation, final approval of the manuscript and follow up.

Fahmi H. Kakamad, Rawezh Q. Salih, Shvan H. Mohammed: Writing the manuscript, final approval of the manuscript and follow up.

Imad H. Habibulla, Karzan Mohammed, Suhaib H. Kakamad, Berwn A. Abdulla, Hiwa O. Baba, Dahat A. Hussein: literature review, final approval of the manuscript.

## Registration of research studies

Not applicable

## Guarantor

Fahmi Hussein Kakamad.

## Provenance and peer review

Not commissioned, externally peer-reviewed.
